# Mechanical Power Correlates With Lung Inflammation Assessed by Positron-Emission Tomography in Experimental Acute Lung Injury in Pigs

**DOI:** 10.3389/fphys.2021.717266

**Published:** 2021-11-22

**Authors:** Martin Scharffenberg, Jakob Wittenstein, Xi Ran, Yingying Zhang, Anja Braune, Raphael Theilen, Lorenzo Maiello, Giulia Benzi, Thomas Bluth, Thomas Kiss, Paolo Pelosi, Patricia R. M. Rocco, Marcus J. Schultz, Jörg Kotzerke, Marcelo Gama de Abreu, Robert Huhle

**Affiliations:** ^1^Department of Anesthesiology and Intensive Care Medicine, Pulmonary Engineering Group, University Hospital Carl Gustav Carus, Technische Universität Dresden, Dresden, Germany; ^2^Department of Intensive Care, Chongqing General Hospital, University of Chinese Academy of Sciences, Chongqing, China; ^3^Department of Anesthesiology, Affiliated Hospital of Southwest Medical University, Luzhou, China; ^4^Department of Nuclear Medicine, University Hospital Carl Gustav Carus, Technische Universität Dresden, Dresden, Germany; ^5^Anesthesia and Critical Care, San Martino Policlinico Hospital, IRCCS for Oncology and Neurosciences, Genoa, Italy; ^6^Department of Clinical and Biological Sciences, Service of Anesthesia and Intensive Care, Ospedale di Circolo e Fondazione Macchi, University of Insubria, Varese, Italy; ^7^Department of Anaesthesiology, Intensive-, Pain- and Palliative Care Medicine, Radebeul Hospital, Academic Hospital of the Technische Universität Dresden, Radebeul, Germany; ^8^Department of Surgical Sciences and Integrated Diagnostics, University of Genoa, Genoa, Italy; ^9^Laboratory of Pulmonary Investigation, Carlos Chagas Filho Institute of Biophysics, Federal University of Rio de Janeiro, Rio de Janeiro, Brazil; ^10^Department of Intensive Care and Laboratory of Experimental Intensive Care and Anaesthesiology, Academic Medical Center, University of Amsterdam, Amsterdam, Netherlands; ^11^Department of Intensive Care and Resuscitation, Anesthesiology Institute, Cleveland Clinic, Cleveland, OH, United States; ^12^Department of Outcomes Research, Anesthesiology Institute, Cleveland Clinic, Cleveland, OH, United States

**Keywords:** mechanical ventilation, acute respiratory distress syndrome, ARDS, ventilator- induced lung injury, VILI, mechanical power, pulmonary neutrophilic inflammation, ^18^F-FDG

## Abstract

**Background:** Mechanical ventilation (MV) may initiate or worsen lung injury, so-called ventilator-induced lung injury (VILI). Although different mechanisms of VILI have been identified, research mainly focused on single ventilator parameters. The mechanical power (MP) summarizes the potentially damaging effects of different parameters in one single variable and has been shown to be associated with lung damage. However, to date, the association of MP with pulmonary neutrophilic inflammation, as assessed by positron-emission tomography (PET), has not been prospectively investigated in a model of clinically relevant ventilation settings yet. We hypothesized that the degree of neutrophilic inflammation correlates with MP.

**Methods:** Eight female juvenile pigs were anesthetized and mechanically ventilated. Lung injury was induced by repetitive lung lavages followed by initial PET and computed tomography (CT) scans. Animals were then ventilated according to the acute respiratory distress syndrome (ARDS) network recommendations, using the lowest combinations of positive end-expiratory pressure and inspiratory oxygen fraction that allowed adequate oxygenation. Ventilator settings were checked and adjusted hourly. Physiological measurements were conducted every 6 h. Lung imaging was repeated 24 h after first PET/CT before animals were killed. Pulmonary neutrophilic inflammation was assessed by normalized uptake rate of 2-deoxy-2-[^18^F]fluoro-D-glucose (K_iS_), and its difference between the two PET/CT was calculated (ΔK_iS_). Lung aeration was assessed by lung CT scan. MP was calculated from the recorded pressure–volume curve. Statistics included the Wilcoxon tests and non-parametric Spearman correlation.

**Results:** Normalized ^18^F-FDG uptake rate increased significantly from first to second PET/CT (*p* = 0.012). ΔK_iS_ significantly correlated with median MP (ρ = 0.738, *p* = 0.037) and its elastic and resistive components, but neither with median peak, plateau, end-expiratory, driving, and transpulmonary driving pressures, nor respiratory rate (RR), elastance, or resistance. Lung mass and volume significantly decreased, whereas relative mass of hyper-aerated lung compartment increased after 24 h (*p* = 0.012, *p* = 0.036, and *p* = 0.025, respectively). Resistance and PaCO_2_ were significantly higher (*p* = 0.012 and *p* = 0.017, respectively), whereas RR, end-expiratory pressure, and MP were lower at 18 h compared to start of intervention.

**Conclusions:** In this model of experimental acute lung injury in pigs, pulmonary neutrophilic inflammation evaluated by PET/CT increased after 24 h of MV, and correlated with MP.

## Introduction

Mechanical ventilation (MV) is often life-saving in critically ill patients with acute respiratory failure and/or acute respiratory distress syndrome (ARDS) (Bellani et al., 2016). However, MV may lead to ventilator-induced lung injury (VILI) (Dreyfuss and Saumon, 1998). Different mechanisms of VILI have been identified so far. High distending pressures may promote baro- and volutrauma, whereas repetitive aeration and collapse of alveoli may induce atelectrauma (Güldner et al., 2016). Inhomogeneous lung aeration can further aggravate mechanical stress and lung injury (Mead et al., 1970). Although certain measures to prevent VILI have been established (Acute Respiratory Distress Syndrome Network Brower et al., 2000; Amato et al., 2015), e.g., limitation of tidal volume (V_T_) or airway plateau and driving pressures, discussion about adequate levels of positive end-expiratory pressure (PEEP) is ongoing, and the interplay among parameters is complex (Battaglini et al., 2021). Irrespectively of specific parameters, mechanical energy is inevitably transferred to the respiratory system in every single MV cycle, resulting in transferred mechanical power (MP) when multiplied with respiratory rate (RR). Although it has been known that the transferred energy, or power, is partly restored and dissipated in the respiratory system (Sassoon and Mahutte, 1998; Guttmann, 2010), the concept gained new attention recently when MP was proposed as the main determinant of VILI (Cressoni et al., 2016; Gattinoni et al., 2016), which is still under development (Huhle et al., 2018). Although recent research mainly focused on single ventilator parameters as VILI determinants, MP may summarize the potentially damaging effects of different parameters in one single variable. Yet, MP was shown to be associated with different characteristics of experimental lung injury, i.e., radiological signs of lung edema, lung wet/dry ratio, and histological features (Cressoni et al., 2016; Collino et al., 2019; Vassalli et al., 2020). In retrospective clinical trials, MP was associated with mortality in critically ill patients (Serpa Neto et al., 2018; Costa et al., 2021). However, its effects on the pulmonary neutrophilic inflammation as assessed by positron-emission tomography (PET)/computed tomography (CT) have not been determined yet, though neutrophilic inflammation is a mainstay in ARDS pathogenesis (Grommes and Soehnlein, 2011). In this study, we aimed to investigate the applied resulting MP and the neutrophilic pulmonary inflammation in a clinically relevant model of acute lung injury in pigs ventilated with fixed combinations of PEEP and inspiratory oxygen fractions (F_I_O_2_), as recommended by the ARDS network’s low PEEP table (Brower et al., 2004). We hypothesized that neutrophilic inflammation correlates with MP.

## Materials and Methods

The Institutional Animal Care and Welfare Committee and the Government of the State of Saxony, Germany, approved the study protocol (file 25-5131/474/31; 27.09.2019; Dr. B. Langen, Landesdirektion Sachsen). Animals received humane care according to German law and the Principles of Laboratory Animal Care formulated by the National Society for Medical Research and the United States National Academy of Sciences Guide for the Care and Use of Laboratory Animals, and the Animal Research: Reporting of *in vivo* Experiments guidelines were followed.

### Animal Preparation and Mechanical Ventilation

Eight female landrace pigs (35–51 kg) were intravenously anesthetized (ketamine, 15 mg/kg/h; midazolam, 1 mg/kg/h), orotracheally intubated, and mechanically ventilated. The following initial settings were used: Volume-controlled ventilation, tidal volume (V_T_) 6 ml/kg, PEEP 5 cmH_2_O, inspiratory to expiratory ratio (I:E) 1:1, inspired fraction of oxygen (F_I_O_2_) 1.0, RR adjusted to normocapnia, and inspiratory flow 35 L/min (Evita XL, Dräger, Lübeck, Germany). V_T_ was reduced if airway plateau pressure (Pplat) was ≥30 cmH_2_O. The right carotid artery, jugular vein, and urinary bladder were catheterized under sterile conditions. A pulmonary artery thermodilution catheter and an esophageal balloon catheter (Cooper Surgical, Trumbull, CT, United States; filling volume 0.5 ml) were introduced. The correct position of the balloon catheter was confirmed as described elsewhere (Lanteri et al., 1994). Animals were paralyzed (atracurium, 3 mg/kg/h) throughout the whole experiment and received a balanced electrolyte infusion of 10 ml/kg/h during preparations and 4 ml/kg/h during intervention time, respectively. The mean arterial pressure (MAP) was kept >60 mmHg by means of norepinephrine and colloid infusion, as appropriate.

### Lung Injury

Following instrumentation, lung injury was induced by repetitive lung lavage with warmed 0.9 % saline in a prone and supine position (37°C; 35 ml/kg; lavage pressure approximately 30 cmH_2_O), until PaO_2_/F_I_O_2_ was <200 mmHg for at least 30 min.

### Experimental Protocol and Intervention Time

**FIGURE 1:**
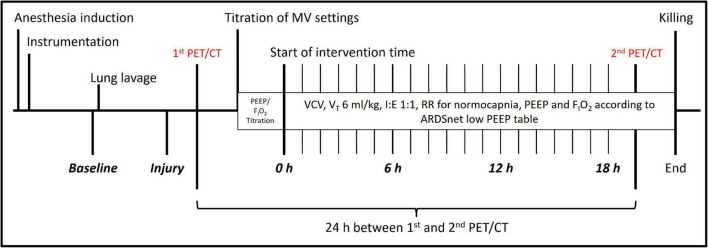
Experimental time course. Experimental time course. PEEP, positive end-expiratory pressure; F_I_O_2_, inspiratory fraction of oxygen; VCV, volume-controlled ventilation; I:E, inspiratory to expiratory time ratio; RR, respiratory rate; ARDSnet, acute respiratory distress syndrome network.

The sequence of events is depicted in Figure 1, which represents a subprotocol of a larger study on the effects of different MV on lung inflammation and function. After instrumentation and induction of lung injury, PET and CT scans were obtained under baseline MV settings, but PEEP of 10 cmH_2_O. Later, MV was adjusted according to the ARDS network recommendations (Brower et al., 2004), as follows: volume-controlled ventilation, V_T_ 6 ml/kg, I:E 1:1, RR adjusted to normocapnia, and flow 35 L/min. The lowest possible fixed PEEP and F_I_O_2_ combination were applied according to the low PEEP table (Brower et al., 2004) to keep PaO_2_ between 55 and 80 mmHg. Lung recruitment maneuvers were not applied. Ventilator settings were titrated within 30 min, and interventional time was started afterwards. Settings were checked hourly and adjusted if necessary. PET/CT lung imaging was repeated 24 h after first PET/CT, and animals were killed by intravenous bolus injection of 2 g thiopental and 50 ml 1 M potassium chloride.

### Measurements

We recorded physiological variables and respiratory signals before (Baseline) and after inducing lung injury (Injury), at the start of intervention time (0 h) and every 6 h thereafter (6, 12, and 18 h), respectively. Blood gasses were analyzed using a commercially available device (ABL80, Radiometer, Brønshøj, Denmark) and cardiac output (CO) was determined by thermodilution method (MP70, Philips Healthcare, Eindhoven, the Netherlands). Stroke volume (SV) was calculated by dividing CO by heart rate (HR).

### Respiratory Signals and Calculations

**FIGURE 2:**
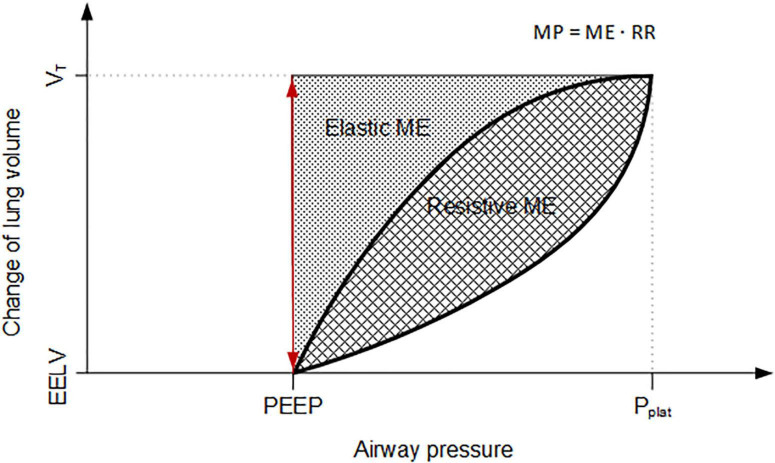
Schematic pressure–volume curve for calculation of mechanical energy. Schematic visualization of changing lung volume and pressure per respiratory cycle, constituting the pressure–volume curve. MP, mechanical power; ME, mechanical energy; RR, respiratory rate; PEEP, positive end-expiratory pressure; P_plat_, plateau airway pressure; V_T_, tidal volume; EELV, end-expiratory lung volume.

Respiratory signals were recorded using a LabVIEW-based software (National Instruments, Austin, TX, United States) and analyzed semi-automatically (MATLAB, R2019a, The MathWorks Inc., Natick, MA, United States). Airway (P_aw_) and esophageal pressure (P_eso_) were measured using differential pressure transducers (Sensortechnics GmbH, Puchheim, Germany) at the endotracheal tube and the balloon catheter, respectively. Furthermore, air flow and airway pressure signals were recorded from the ventilator using a serial interface. The ventilator’s airway pressure signal was used for the offline synchronization of all recorded signals. Peak (P_peak_) and mean (P_mean_) airway pressure were determined as Paw maximum and average during one respiratory cycle from the respiratory tracings, respectively. Driving pressure (ΔP) and transpulmonary pressure (P_trans_) were calculated as P_plat_−PEEP and P_aw_−Peso, respectively. Transpulmonary driving pressure (ΔP_trans_) was calculated as plateau−minimal P_trans_. Elastance (E) and resistance (R) were derived by fitting the equation of motion to the acquired respiratory signals (multiple linear regression). The percentage of volume-dependent elastance (%E_2_) was determined as described elsewhere (Kano et al., 1994; Carvalho et al., 2013). MP was calculated using the recorded respiratory signals, which included continuous recordings of airway pressure and flow. V_T_ was calculated from the latter. First, mechanical energy by breath (ME) is defined as the numerical integral of the airway pressure and volume changes (Huhle et al., 2018), constituting the tidal pressure–volume curve (PV curve), see Figure 2. Second, ME was multiplied by RR to achieve MP. During each breath, energy is spend to overcome resistance and elastic forces; thus, the corresponding components, i.e., resistive and elastic power, can be calculated. Alveolo-arterial oxygen difference (AaDO_2_) and venous admixture were calculated using standard formulas.

### PET and CT

The PET-based assessment of tissue uptake rate of the radio-tracer 2-deoxy-2-[^18^F]fluoro-D-glucose (^18^F-FDG) served as surrogate for neutrophilic pulmonary inflammation (Musch et al., 2007; Wadsak and Mitterhauser, 2010) and was measured by dynamic ^18^F-FDG PET scans (Biograph Vision 600 PET/CT, Siemens Healthineers, Knoxville, TN, United States), as described elsewhere (Braune et al., 2019; Kiss et al., 2019; Wittenstein et al., 2020). In brief, a static low-dose CT scan (attenuation-correction computed tomography, ACCT, approximately 5 s) for attenuation correction of the following PET scan was performed at mean airway pressure hold and followed by injection of ^18^F-FDG (∼200 MBq) and dynamic PET scan (26 cm craniocaudal field of view, 75 min) before the start of the intervention time. Blood samples were collected throughout the dynamic PET scan to assess the tracer’s plasma activity (cross-calibrated gamma counter). These lung imaging procedures were repeated 24 h after first PET/CT. The dynamic PET scans were reconstructed using an OSEM 3D iterative reconstruction, applying point spread function (PSF) and time of flight and correcting for attenuation and scatter, into an image matrix size of 440 × 440, resulting in a voxel size of 1.65 mm × 1.65 mm × 2.0 mm. ACCTs co-registered to PET scans were used to semi-automatically create lung masks and calculate gas fractions by F_Gas_ = CT number [Hounsfield unit HU]/−1000 (Gattinoni et al., 2001). The ^18^F-FDG uptake rate (K_i_) was calculated using the Patlak model using the software Rover (ABX GmbH, Radeberg, Germany) (Schroeder et al., 2011; Torigian et al., 2009). K_i_ was then normalized to the tissue fraction (K_iS_) (Wittenstein et al., 2020) as follows:


(1) $$K_{i\,S}=\frac{K\,i}{F_{T\,i\,s\,s\,u\,e}}=\frac{K\,i}{\left(1-F_{G\,a\,s}-F_{B\,l\,o\,o\,d}\right)},$$

where, K_iS_ stands for tissue-normalized ^18^F-FDG uptake rate, F_Gas_ for gas fraction, and F_Blood_ for blood fraction derived using the Sokoloff model (Schroeder et al., 2011). Differences between lung imaging data before and after intervention time (second PET/CT – first PET/CT) were calculated using median over the respective semi-automatically segmented lung regions of interest (ROIs). ROIs were segmented using a semi-automatic approach consisting of automated segmentation using a deep convolutional neural network algorithm trained on static CT scans from previous animal studies, followed by manual correction by two independent, trained physicians. Hyper (<−900 HU), normal (−900 to −500 HU), poor (−500 to 100 HU), and non-aerated (>−100 HU) lung compartment were computed as described elsewhere based on ACCT data (Hedenstierna et al., 1989).

### Statistical Analysis

This study was exploratory in nature, and thus, its sample size was based on the experience of our group with previous studies on MV and neutrophilic inflammation (Kiss et al., 2019; Wittenstein et al., 2020). However, we defined K_iS_ as the primary outcome. Data are presented as median and interquartile range (IQR), if not stated differently. Statistical differences of variables between baseline and injury and between 0 and 18 h were analyzed using a paired non-parametric test (Wilcoxon test, asymptotic significance, two-sided). Association between median variables and inflammation was assessed using non-parametric spearman correlation. Statistics were performed using SPSS (version 27, IBM Corp., Armonk, NY, United States). Significance was accepted at *p* < 0.05. Graphs were created using GraphPad Prism (version 6.0, GraphPad Software, San Diego, CA, United States).

## Results

### General Results

All eight animals were included in the analysis. Median body weight was 47.7 (8.0) kg. Animals received median 8 (3) lavages to reach the lung injury criteria. The median intervention time, i.e., time from start of intervention time to completion of last lung imaging, was 22.75 h (27 min). In total, animals received median doses of 174 (4) ml/kg of crystalloids, and 0.144 (1.863) μg/kg of norepinephrine during the intervention time. Median cumulative urine output was 67 (12) ml/kg.

### Respiratory Variables

**FIGURE 3:**
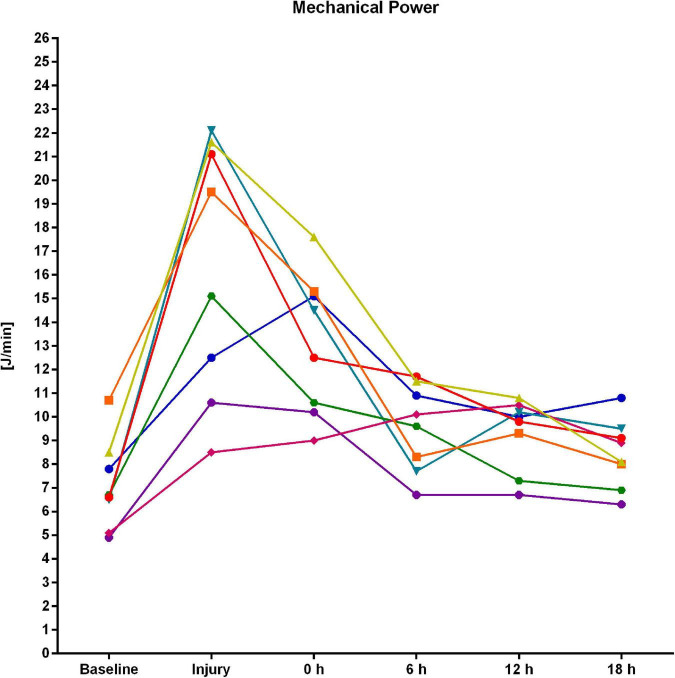
Individual time course of mechanical power derived from the pressure–volume curve for each of the eight experiments.

Table 1 shows the respiratory variables and Figure 3 depicts the individual time course of MP for each experiment. After induction of experimental lung injury, RR, P_peak_, P_plat_, ΔP, peak P_trans_, ΔP_trans_, resistance, elastance, %E_2_, MP, elastic MP, resistive MP, AaDO_2_, and venous admixture were significantly higher as compared to baseline. PaO_2_ and PaO_2_/F_I_O_2_ were significantly lower after injury than at baseline, whereas other variables did not differ significantly between these two pre-intervention time points. After 18 h of intervention, resistance and PaCO_2_ were significantly increased, whereas RR, PEEP, MP, and elastic and resistive MP were significantly lower as compared to the start of the intervention. The remaining variables did not differ significantly between begin and end of the intervention time.

**TABLE 1:** Respiratory variables.

Variable	Baseline	Injury	0 h	6 h	12 h	18 h	BL vs. Injury *p*	0 h vs. 18 h *p*
Tidal volume (ml/kg)	6.5 (0.2)	6.6 (0.2)	6.5 (0.2)	6.5 (0.2)	6.5 (0.2)	6.5 (0.3)	0.327	0.484
Respiratory rate (1/min)	25 (7)	31 (9)	31 (5)	22 (6)	19 (5)	17 (3)	**0.018**	**0.012**
F_I_O_2_ (0.0–1.0)	1.00 (0.03)	1.00 (0.00)	0.32 (0.09)	0.32 (0.01)	0.32 (0.01)	0.32 (0.02)	0.109	0.438
P_peak_ (cmH_2_O)	19.2 (2.8)	36.9 (6.6)	28.9 (3.8)	29.3 (2.7)	30.6 (2.9)	30.1 (4.5)	**0.012**	0.889
P_plat_ (cmH_2_O)	13.8 (1.1)	30.2 (4.9)	24.5 (2.3)	23.1 (3.9)	23.1 (3.5)	22.9 (3.1)	**0.012**	0.093
ΔP (cmH_2_O)	8.8 (0.9)	25.3 (5.2)	19.1 (3.4)	18.1 (3.9)	18.3 (3.6)	18.2 (3.0)	**0.012**	0.779
PEEP_set_ (cmH_2_O)	5.0 (0)	5.0 (0)	5.0 (0)	5.0 (0)	5.0 (0)	5.0 (0)	–	–
PEEP_measured_ (cmH_2_O)	5.1 (0.3)	5.0 (0.2)	5.1 (0.1)	4.9 (0.2)	4.8 (0.2)	4.9 (0.1)	0.352	**0.011**
Resistance (cmH_2_O s/l)	10.5 (4.0)	14.9 (1.8)	12.4 (2.0)	15.4 (2.3)	16.6 (3.4)	16.6 (5.6)	**0.012**	**0.012**
Elastance (cmH_2_O/l)	26.2 (4.9)	83.0 (20.6)	57.3 (15.0)	56.3 (12.0)	56.3 (10.7)	54.7 (9.3)	**0.012**	0.484
%E_2_ (%)	−37.8 (0.4)	−12.6 (9.4)	−24.6 (3.5)	−28.3 (4.1)	−v28.6 (3.1)	−29.5 (4.0)	**0.012**	0.069
MP (J/min)	6.6 (2.9)	17.3 (10.4)	13.5 (4.9)	9.8 (3.5)	9.9 (2.6)	8.5 (2.2)	**0.012**	**0.012**
MP elastic (J/min)	4.5 (1.9)	11.8 (7.1)	9.1 (3.3)	6.7 (2.5)	6.7 (1.8)	5.7 (1.5)	**0.012**	**0.012**
MP resistive (J/min)	2.2 (1.0)	5.5 (3.3)	4.4 (1.6)	3.1 (1.0)	3.2 (0.8)	2.7 (0.7)	**0.012**	**0.017**
P_trans_ peak (cmH_2_O)	8.8 (4.2)	25.1 (4.8)	18.4 (3.2)	17.9 (3.4)	18.6 (4.0)	18.7 (2.4)	**0.012**	0.889
P_trans_ endex (cmH_2_O)	−3.5 (2.7)	−4.4 (2.0)	−3.9 (2.9)	−4.8 (2.8)	−4.3 (2.6)	−4.4 (0.8)	0.123	0.123
ΔP_trans_ (cmH_2_O)	12.0 (4.0)	29.3 (4.5)	21.6 (4.5)	22.3 (3.3)	23.4 (2.3)	23.2 (3.7)	**0.012**	0.327
PaO_2_ (mmHg)	530.3 (277.3)	100.5 (73.1)	106.0 (50.8)	109.5 (17.3)	110.5 (23.5)	105.5 (19.1)	**0.012**	0.327
PaCO_2_ (mmHg)	55.7 (5.6)	50.2 (10.4)	46.4 (16.9)	54.0 (9.3)	51.3 (5.1)	60.2 (4.4)	0.161	**0.017**
pHa	7.37 (0.08)	7.37 (0.07)	7.42 (0.11)	7.40 (0.04)	7.42 (0.03)	7.40 (0.05)	0.528	0.236
PaO_2_/F_I_O_2_ (mmHg)	528 (265)	101 (73)	301 (125)	352 (44)	349 (74)	320 (91)	**0.012**	0.208
AaDO_2_ (mmHg)	115.1 (241.4)	549.8 (93.6)	79.4 (40.4)	45.0 (32.7)	49.0 (37.2)	50.8 (38.1)	**0.012**	0.036
Venous admixture (%)	8.8 (37.1)	47.6 (16.9)	21.1 (14.0)	14.7 (3.2)	10.9 (7.9)	14.6 (13.2)	**0.012**	0.327

*Median (IQR); BL: baseline; V_T_, tidal volume; F_I_O_2_, inspiratory oxygen fraction; P_peak_, peak airway pressure; P_plat_, plateau airway pressure; ΔP, driving pressure; PEEP, positive end-expiratory pressure; %E_2_, volume-dependent elastance; MP, mechanical power calculated from pressure–volume curve; P_trans_ peak, peak transpulmonary pressure; P_trans_ endex, end-expiratory transpulmonary pressure; ΔP_trans_, transpulmonary driving pressure; PaO_2_, arterial partial pressure of oxygen; PaCO_2_, arterial partial pressure of carbon dioxide; pHa, arterial pH value; PaO_2_/F_I_O_2_, Horovitz index; AaDO_2_, alveolo-arterial oxygen difference. Comparisons baseline vs. injury and 0 h vs. 18 h: Wilcoxon test, asymptotic significance, two-sided, significance accepted at p < 0.05. Bold p-values show significant differences (all p-values below 0.05).*

### Neutrophilic Inflammation

**FIGURE 4:**
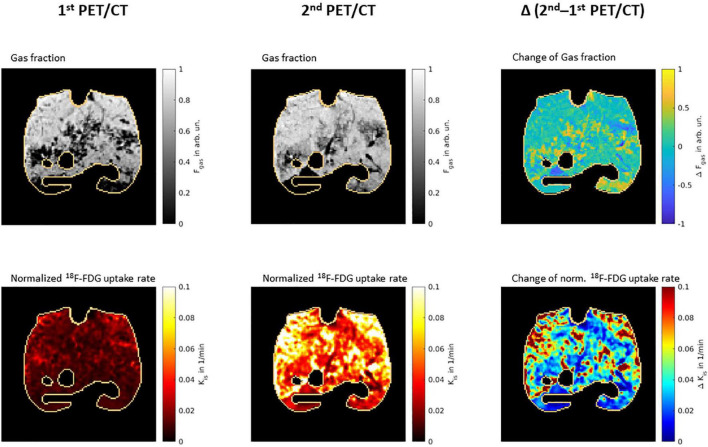
Representative PET/CT scans. Representative PET/CT scans from one representative animal, showing gas fraction **(upper row)** and normalized ^18^F-FDG uptake rate **(lower row)** from first **(left column)** and second lung scans **(middle column)**. The right column shows the difference between second and first scan after co-registration of ACCTs using Elastix Library. The lung masks are outlined in copper color. F_gas_, gas fraction; arb. un., arbitrary units; ^18^F-FDG, 2-deoxy-2-[^18^F]fluoro-D-glucose; K_iS_, normalized uptake rate of 2-deoxy-2-[^18^F]fluoro-D-glucose.

**FIGURE 5:**
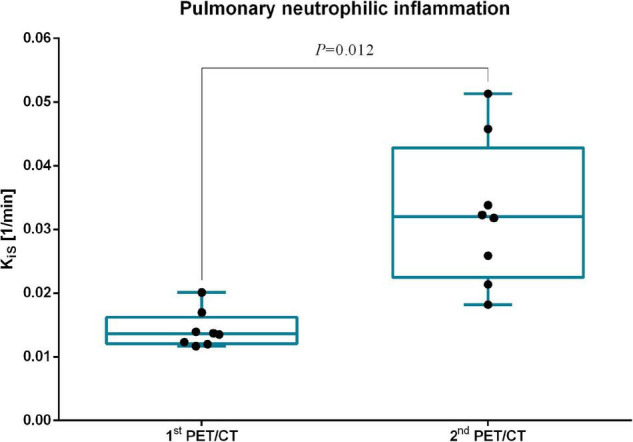
Normalized ^18^F-FDG uptake rate (K_iS_) before and after intervention time. Normalized ^18^F-FDG uptake rate (K_iS_) before and after intervention time. PET/CT, positron-emission tomography; CT, computed tomography.

**FIGURE 6:**
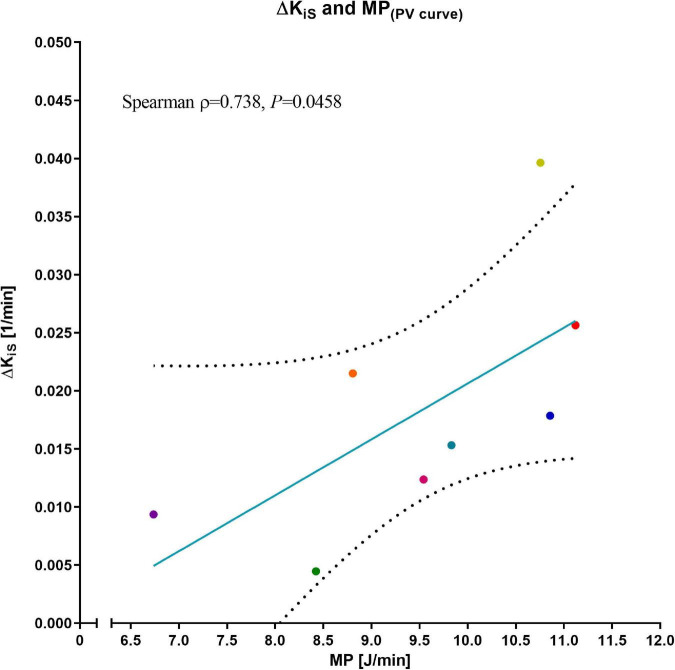
Correlation of ΔK_iS_ and mechanical power. Normalized ^18^F-FDG uptake rate, expressed as difference between the two PET/CT scans (ΔK_iS_), and mechanical power (MP) as assessed from the pressure–volume curve; dotted lines: 95 % confidence band of the best-fit line. Correlation was assessed as non-parametric bivariate correlation (Spearman). Colors represent corresponding animals from Figure 3.

Representative PET/CT scans are presented in Figure 4. As compared to the first PET/CT, K_iS_ was significantly higher after 24 h (second PET/CT 0.0320 (0.0203) min^–1^ vs. first PET/CT 0.0136 (0.0041) min^–1^; *p* = 0.012; Figure 5). ΔK_iS_ significantly correlated with median MP derived from the PV curve (Figure 6) and with the median elastic and resistive MP components (Table 2). In contrast, the other respiratory variables did not correlate with ΔK_iS_ (Table 2).

**TABLE 2:** Correlation between respiratory variables and ΔK_iS_.

Variable	Spearman ρ	*P*
MP (J/min)	0.738	**0.037**
MP elastic (J/min)	0.738	**0.037**
MP resistive (J/min)	0.738	**0.037**
P_peak_ (cmH_2_O)	0.405	0.320
P_plat_ (cmH_2_O)	0.310	0.456
ΔP (cmH_2_O)	0.310	0.456
ΔP_trans_ (cmH_2_O)	0.095	0.823
RR (1/min)	−0.108	0.798
Elastance (cmH_2_O/l)	−0.357	0.385
Resistance (cmH_2_O s/l)	0.452	0.260
%E_2_ (%)	0.262	0.531

*Spearman coefficient ρ for correlation of displayed respiratory variables with ΔK_iS_. MP, mechanical power obtained from pressure–volume curves; P_peak_, peak airway pressure; P_plat_, plateau airway pressure; ΔP, driving pressure; ΔP_trans_, transpulmonary driving pressure; RR, respiratory rate; %E_2_, percentage of volume-dependent elastance. Statistical test two-sided, significance accepted at p < 0.05. Bold p-values show significant differences (all p-values below 0.05).*

### Lung Aeration

**FIGURE 7:**
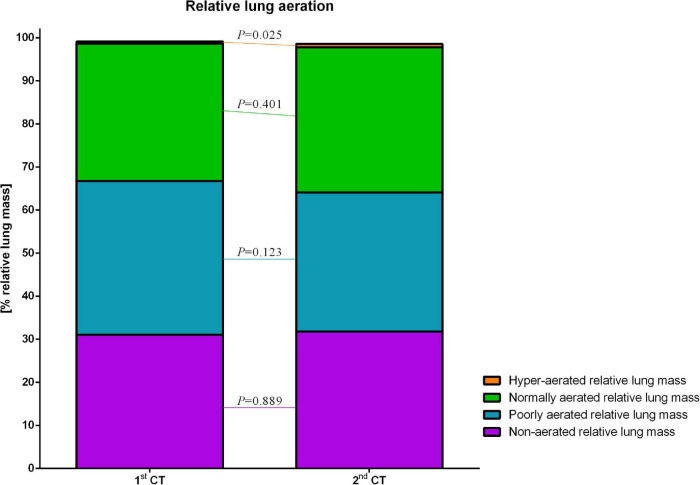
Relative lung aeration. Different degrees of lung aeration, expressed as % relative lung mass.

Both median total lung mass and total pulmonary gas volume significantly decreased from first to second lung imaging [854.1 (177.2) vs. 635.6 (121.9) g, *p* = 0.012; and 850.7 (143.2) vs. 780.2 (185.9) ml, *p* = 0.036, respectively]. In relation to total lung mass in the corresponding PET/CT, the relative mass of hyper-aerated compartment of the lung increased significantly from the first to the second PET/CT scan [0.48 (0.45)% vs. 0.75 (0.64)%, *p* = 0.025]. The relative mass of normally, poorly, and non-aerated lung mass did not differ significantly between the scan before and after the intervention time (Figure 7). The net relative lung aeration, defined as sum of poorly, normally, and hyper-aerated lung compartments, did not differ between first and second PET/CT [69.4 (11.1)% vs. 67.1 (15.8)%, *p* = 0.889].

### Hemodynamic Variables

Table 3 depicts the hemodynamic variables. As compared with baseline, MAP, mean pulmonary arterial pressure (MPAP), pulmonary capillary wedge pressure, and central venous pressure were significantly higher after induction of lung injury, whereas HR, SV, and CO did not differ significantly. The majority of hemodynamic variables did not differ significantly between start and end of intervention time, despite cardiac SV, which was significantly higher after 18 h of intervention time.

**TABLE 3:** Hemodynamic variables.

Variable	Baseline	Injury	0 h	6 h	12 h	18 h	BL vs. Injury *p*	0 h vs. 18 h *p*
MAP (mmHg)	72 (11)	85 (15)	81 (16)	75 (13)	74 (7)	76 (13)	**0.011**	0.127
MPAP (mmHg)	17 (4)	33 (4)	25 (5)	24 (5)	23 (6)	24 (10)	**0.012**	0.526
PCWP (mmHg)	9 (4)	11 (5)	7 (2)	8 (3)	8 (3)	8 (3)	**0.017**	0.053
CVP (mmHg)	6 (4)	9 (4)	5 (4)	5 (2)	6 (4)	6 (2)	**0.011**	0.139
HR (1/min)	96 (17)	94 (22)	120 (28)	98 (20)	94 (9)	104 (30)	0.092	0.058
SV (ml)	54 (10)	59 (12.0)	50 (15)	63 (19)	63 (9)	68 (13)	0.036	**0.012**
CO (l/min)	5.3 (1.0)	5.8 (1.8)	6.2 (2.4)	6.1 (2.6)	5.9 (1.9)	7.1 (2.1)	0.612	0.093

*Median (IQR); BL, baseline; MAP, mean arterial pressure; MPAP, mean pulmonary arterial pressure; PCWP, pulmonary capillary wedge pressure; CVP, central venous pressure; HR, heart rate; SV, stroke volume; CO, cardiac output; comparisons baseline vs. injury and 0 h vs. 18 h, Wilcoxon test, asymptotic significance, two-sided, significance accepted at p < 0.05. Bold p-values show significant differences (all p-values below 0.05).*

## Discussion

The main findings of the present study are in pigs ventilated mechanically according to the low PEEP table of the ARDS network: (1) pulmonary neutrophilic inflammation, as assessed by PET/CT, increased significantly over time; (2) among all ventilatory variables investigated, only MP and its elastic and resistive components showed a positive correlation with neutrophilic inflammation; and (3) global pulmonary gas volume decreased whereas hyper-aerated relative lung mass increased over time.

To the best of our knowledge, this is the first experimental study that prospectively investigated the relationship between MP and pulmonary neutrophilic inflammation as assessed with PET/CT. Previous studies were limited to a less sensitive assessment of VILI, including radiographic evidence of pulmonary edema, cumulative histological scores, and lung wet/dry ratio (Cressoni et al., 2016; Collino et al., 2019; Vassalli et al., 2020). Neutrophilic infiltration and inflammation is deemed to be a main pathological mechanism in the development and course of ARDS (Zemans et al., 2009; Grommes and Soehnlein, 2011; Matthay and Zemans, 2011).

A particular strength of our study is that we used MV settings of a clinically common MV concept in a model of moderate acute lung injury, rather than intentionally provoking lung injury by unusual or artificial settings or investigating the roles of certain ventilator parameters. Instead, we applied fixed PEEP/F_I_O_2_ combinations according to the ARDS network recommendations (low PEEP table) (Brower et al., 2004). Although large clinical trials did not reveal an outcome advantage of high over low PEEP in patients with ARDS (Brower et al., 2004; Meade et al., 2008; Mercat et al., 2008), MV concepts tolerating formation of atelectasis and moderate hypercapnia in order to avoid aggressive ventilation in terms of high distending pressures are under debate (Pelosi et al., 2018) and clinically favored. Use of the low PEEP table was recently shown to increase survival in patients with ARDS as compared with individualized but higher PEEP (Cavalcanti et al., 2017). In addition, titrating PEEP according to lung morphology did not improve outcome and even increased mortality if morphology was misclassified, respectively. Thus, as proposed earlier (Brower et al., 2004), fixed PEEP/F_I_O_2_ combinations should still be used for patients with ARDS (Chiumello et al., 2014; Battaglini et al., 2021), which justified our selected experimental setting.

The finding that MV at relatively low PEEP increased neutrophilic inflammation is in line with a previous experimental study by our group (Kiss et al., 2019). Therein, PEEP was titrated according to end-expiratory transpulmonary pressure allowing end-expiratory alveolar collapse. This approach was shown to increase neutrophilic inflammation as compared with higher PEEP under controlled MV (Kiss et al., 2019). In the present study, PEEP was titrated according to clinically common oxygenation thresholds, and according to the low PEEP/F_I_O_2_ table from the ARDS Net, yielding PEEP levels even lower than the titrated PEEP in the mentioned previous trial. Thus, it is not surprising that pulmonary neutrophilic inflammation was detected and even increased over time herein. Furthermore, median ΔP was higher than 15 cmH_2_O throughout the intervention time, which was postulated as a threshold for increased risk of mortality in a retrospective clinical analysis of patients with ARDS (Amato et al., 2015). Although existing meta-analyses are ambiguous, our finding of increased pulmonary neutrophilic inflammation might be recognized as contradictory to the observation that survival in ARDS may be higher with lower PEEP (Cavalcanti et al., 2017). However, in general, clinical outcomes are influenced by many effects, e.g., disease severity, comorbidities, ventilation settings potentially inducing VILI, and hemodynamics. One could hypothesize that there is a similar inflammatory response in patients with ARDS undergoing similar ventilator settings, but that other effects in the recent clinical trials, e.g., impaired hemodynamics, affect outcomes majorly as well. Our study cannot assess how clinically relevant our observed pulmonary inflammation is and direct comparison with the recent large clinical trials must be done cautiously. However, we focused on an established mechanism in ARDS pathophysiology, the neutrophilic inflammation, which served as VILI surrogate in many high-quality experimental studies.

Despite the time point directly after starting the intervention time (0 h), median MP was always lower than the initially postulated VILI threshold of 12 J/min (Cressoni et al., 2016). However, it was high at Injury and the start of intervention time (0 h). Thus, it is not surprising that there is neutrophilic inflammation, though MP was relatively low during intervention time. This is also in line with the findings from another experimental study, where the lowest power was also associated with histological evidence of VILI (Collino et al., 2019). In a third experimental investigation, cumulative histological lung injury did not differ between MP around 14 and 29 J/min (Vassalli et al., 2020). In this regard, two major considerations have to be made. First, absolute MP values depend on the way of calculation. We used the respiratory tracings to assess MP, whereas different MP formulas have been postulated since the seminal publication in 2016 (Giosa et al., 2019). The major criticism regarding the initial formula was that there is incorrect mathematical modeling of the role of PEEP (Huhle et al., 2018), whereas others tried to reduce the formula’s complexity (Marini and Jaber, 2016; Giosa et al., 2019). This issue needs further investigation. Second, different experimental models and settings preclude from direct comparison of certain MP values and their respective associated results.

Although ΔP, E, and V_T_ did not change and R increased during the intervention time, both RR and MP decreased, i.e., changed in the same direction. Of course, RR contributes linearly to MP; thus, RR and MP are closely linked mathematically. Still, one could hypothesize that it could be mainly RR contributing to VILI in our study. In fact, RR was associated with lung injury under certain circumstances (Rich et al., 2003). However, only MP correlated significantly with ΔK_iS_ in our study, whereas RR did not. The finding that MP but not is determinants, i.e., ΔP and RR, were associated with pulmonary neutrophilic inflammation may strengthen the potential role of MP regarding VILI as a unifying variable, summarizing the burden resulting from the clinically selected ventilator settings. For the first time, our study combined concepts of pulmonary neutrophilic inflammation and MP. Although the first is generally considered a cellular quantity indicative of VILI, the latter is discussed as a VILI determinant. MP depends on a number of factors including ventilator settings (ΔP, V_T_, RR, etc.) and respiratory mechanics (R and E). Although driving pressure may reflect the mechanical burden, i.e., stress per breath alone, it does not reflect how often this mechanical stress is applied per unit of time. RR quantifies mechanical stress applied per unit of time. In a gross approximation, MP is the product of both RR and driving pressure. Consequently, although neither driving pressure nor RR are associated with neutrophilic inflammation, MP may still do because both do not necessarily covariate. In this investigation, we found an association of MP with neutrophilic inflammation supporting the notion that MP may be a determinant of VILI superior to the single respiratory components. Of note, this statistical association does not prove causality. During our intervention time, PEEP decreased significantly. However, its median difference between 0 and 18 h was only 0.2 cmH_2_O, which we would not consider as clinically relevant. Thus, its contribution to the shown decrease of MP may have been negligible.

The CT scans revealed that both the total pulmonary gas volume decreased during the intervention time, whereas the relative mass of lung aeration compartments did not change substantially, despite the significantly increased hyper-aerated compartment. The decreased lung mass may be explained by a decrease of intrapulmonary fluid content, because lung lavage fluid partly remained in the airways and alveoli after induction of lung injury, but was removed by positive pressure ventilation and capillary/lymphatic resorption within the intervention time and before second CT. This is in line with our experiences with the lavage model. The total gas volume decreased most likely because first CT scan was performed at PEEP of 10 cmH_2_O, whereas median PEEP was substantially lower at the moment of second CT (∼5 cmH_2_O). Interestingly, net relative lung aeration (defined as aerated relative lung mass in relation to total lung mass at respective CT scans) did not differ; thus, the atelectatic compartment remained stable. Accordingly, median end-expiratory transpulmonary pressure was negative throughout the intervention time, which promotes end-expiratory lung collapse and atelectasis formation. Furthermore, elastance, ΔP, and transpulmonary driving pressure did not differ significantly between start and end of the intervention time. The significant increase of PaCO_2_ over time could suggest decreasing lung aeration, but venous admixture did not differ between 0 and 18 h. More likely, PaCO_2_ increased because RR was decreased to keep arterial pH in a desired range. Within the aerated lung compartments, only relative hyper-aeration increased significantly. Although this was not accompanied by significant changes of %E_2_, it fits to the finding of increased K_iS_ in the second PET/CT.

As the only hemodynamic variable, SV significantly differed between 0 and 18 h. Most likely, hemodynamics were still impaired at 0 h related to the recent induction of lung injury. Already 6 h later, SV increased and remained stable or even increased until 12 and 18 h, respectively. The latter may reflect further hemodynamic stabilization or recovery, and may be related to the increasing cumulative intravenous fluid administration. In contrast, increased SV could be interpreted as a reaction to the increasing inflammatory status. However, HR, CO, MAP, and the need for norepinephrine did not change significantly, rendering this explanation less likely.

### Possible Clinical Implications of the Findings

Because MP was associated with a major pathological mechanism of lung injury in ARDS, namely neutrophilic infiltration and inflammation, clinicians may consider adjusting MV to reduce MP, whereas providing minimally acceptable gas exchange.

### Limitations

The present study knows limitations. First, this was a relatively small, explorative experimental trial in pigs to investigate prospectively the association of MP with pulmonary neutrophilic inflammation, which limits direct extrapolation to humans. Second, we used an injury model based on lung lavages only, precluding direct translation to different models. Third, we did not provoke different magnitudes of MP, which may limit the extrapolation to other MV settings or concepts, e.g., higher PEEP or open lung approach. However, we aimed to investigate the effects in an experimental model reflecting common clinical settings. Fourth, PEEP differed between the first and the second PET/CT. However, PEEP was adjusted according to defined criteria, did not differ between animals, and may reflect clinically relevant time course. Fifth, we assessed VILI in terms of the metabolic activity as indicated by normalized ^18^F-FDG uptake rate. Although other metabolically active cells may also accumulate this tracer, previous studies identified K_iS_ as a reliable VILI surrogate (Jones et al., 1997; Musch et al., 2007; Costa et al., 2010; Saha et al., 2013). We addressed only the short- to mid-term effects, resembling the very early phase of ARDS. Sixth, we did not evaluate histological VILI features, because other investigators have successfully completed histological analyses previously (Collino et al., 2019; Vassalli et al., 2020). In this regard, our study expands previous trials. Seventh, results are only valid for controlled ventilation, which we used herein. Results may differ in a clinical setting where spontaneous breathing is increasingly accepted nowadays, also within the first 24 h.

## Conclusion

In experimental acute lung injury in pigs, MV according to the recommendations of the ARDS network and using the low PEEP/F_I_O_2_ table resulted in increased PET/CT-derived pulmonary neutrophilic inflammation, which correlated with MP.

## Data Availability Statement

The raw data supporting the conclusions of this article will be made available by the authors, without undue reservation.

## Ethics Statement

The animal study was reviewed and approved by Landesdirektion Sachsen, Referat 25, Chemnitz.

## Author Contributions

MS, JW, RH, JK, PP, MJS, PR, and MG planned and designed the study. MS, JW, RH, XR, YZ, AB, RT, LM, and GB performed the experiments. MS, JW, MG, AB, RH, and JK planned, performed and analyzed the PET/CT image acquisition. MS, JW, RH, XR, YZ, RT, LM, GB, TB, TK, and MG were involved in the data analyses. YZ, RT, LM, and RH performed the lung segmentations (semi-automatic followed by manual correction), image data preprocessing, and co-registration. MS, JW, RH, and MG wrote the manuscript draft. All authors read and approved the submitted manuscript, and agreed to be accountable for the content of the work and its publication.

## Conflict of Interest

MG received consultation fees from Dräger, Ambu, GE Healthcare, and ZOLL. The remaining authors declare that the research was conducted in the absence of any commercial or financial relationships that could be construed as a potential conflict of interest.

## Publisher’s Note

All claims expressed in this article are solely those of the authors and do not necessarily represent those of their affiliated organizations, or those of the publisher, the editors and the reviewers. Any product that may be evaluated in this article, or claim that may be made by its manufacturer, is not guaranteed or endorsed by the publisher.

## Abbreviations

%E_2_: volume-dependent elastance
Δ K_iS_: difference in K_iS_ between first and second lung imaging
Δ P: driving pressure
Δ Ptrans: transpulmonary driving pressure
^18^F-FDG: 2-deoxy-2-[^18^F]fluoro-D-glucose
AaDO_2_: alveolo-arterial oxygen difference
ACCT: attenuation-correction computed tomography
ARDS: acute respiratory distress syndrome
CO: cardiac output
E: elastance
F_I_O_2_: inspiratory oxygen fraction
HU: hounsfield units
HR: heart rate
I:E: inspiratory to expiratory time ratio
IQR: interquartile range
K_iS_: normalized ^18^F-FDG uptake rate
ME: mechanical energy
MP: mechanical power
MV: mechanical ventilation
PaCO_2_: arterial partial pressure of carbon dioxide
PaO_2_: arterial partial pressure of oxygen
PaO_2_/F_I_O_2_: horovitz index
P_aw_: airway pressure
P_peak_: peak airway pressure
PEEP: positive end-expiratory pressure
P_eso_: esophageal pressure
P_mean_: mean airway pressure
P_plat_: mean plateau pressure
R: resistance
RR: respiratory rate
SV: stroke volume
VILI: ventilator-induced lung injury
V_T_: tidal volume.

## References

[B1] Acute Respiratory Distress Syndrome Network, Brower R. G. Matthay M. A. Morris A. Schoenfeld D. Thompson B. T. (2000). Ventilation with lower tidal volumes as compared with traditional tidal volumes for acute lung injury and the acute respiratory distress syndrome. *N. Engl. J. Med.* 342 1301–1308. 10.1056/NEJM200005043421801 10793162

[B2] Amato M. B. P. Meade M. O. Slutsky A. S. Brochard L. Costa E. L. V. Schoenfeld D. A. (2015). Driving pressure and survival in the acute respiratory distress syndrome. *N. Engl. J. Med.* 372 747–755. 10.1056/NEJMsa1410639 25693014

[B3] Battaglini D. Sottano M. Ball L. Robba C. Rocco P. R. M. Pelosi P. (2021). Ten golden rules for individualized mechanical ventilation in acute respiratory distress syndrome. *J. Intens. Med.* Available online at: https://www.sciencedirect.com/science/article/pii/S2667100X21000049 (accessed March 19, 2021). 10.1016/j.jointm.2021.01.003 PMC7919509 36943812

[B4] Bellani G. Laffey J. G. Pham T. Fan E. Brochard L. Esteban A. (2016). Epidemiology, patterns of care, and mortality for patients with acute respiratory distress syndrome in intensive care Units in 50 Countries. *JAMA* 315 788–800. 10.1001/jama.2016.0291 26903337

[B5] Braune A. Hofheinz F. Bluth T. Kiss T. Wittenstein J. Scharffenberg M. (2019). Comparison of static 18F-FDG-PET/CT (SUV, SUR) and dynamic 18F-FDG-PET/CT (Ki) for quantification of pulmonary inflammation in acute lung injury. *J. Nucl. Med.* 60 1629–1634. 10.2967/jnumed.119.226597 31053684

[B6] Brower R. Lanken P. MacIntyre N. Matthay M. Morris A. Ancukiewicz M. (2004). Higher versus lower positive end-expiratory pressures in patients with the acute respiratory distress syndrome. *New Engl. J. Med.* 351 327–336. 10.1056/NEJMoa032193 15269312

[B7] Carvalho A. R. Pacheco S. A. de Souza Rocha P. V. Bergamini B. C. Paula L. F. Jandre F. C. (2013). Detection of tidal recruitment/overdistension in lung-healthy mechanically ventilated patients under general anesthesia. *Anesth Analg.* 116 677–684. 10.1213/ANE.0b013e318254230b 22543064

[B8] Cavalcanti A. B. Suzumura ÉA. Laranjeira L. N. Paisani D. de M. Damiani L. P. (2017). Effect of lung recruitment and titrated positive end-expiratory pressure (PEEP) vs low PEEP on mortality in patients with acute respiratory distress syndrome: a randomized clinical trial. *JAMA* 318 1335–1345. 10.1001/jama.2017.14171 28973363 PMC5710484

[B9] Chiumello D. Cressoni M. Carlesso E. Caspani M. L. Marino A. Gallazzi E. (2014). Bedside selection of positive end-expiratory pressure in mild, moderate, and severe acute respiratory distress syndrome. *Crit. Care Med.* 42 252–264. 10.1097/CCM.0b013e3182a6384f 24196193

[B10] Collino F. Rapetti F. Vasques F. Maiolo G. Tonetti T. Romitti F. (2019). Positive end-expiratory pressure and mechanical power. *Anesthesiology* 130 119–130. 10.1097/ALN.0000000000002458 30277932

[B11] Costa E. L. V. Musch G. Winkler T. Schroeder T. Harris R. S. Jones H. A. (2010). Mild endotoxemia during mechanical ventilation produces spatially heterogeneous pulmonary neutrophilic inflammation in sheep. *Anesthesiology* 112 658–669. 10.1097/ALN.0b013e3181cbd1d4 20179503 PMC2829720

[B12] Costa E. L. V. Slutsky A. Brochard L. J. Brower R. Serpa-Neto A. Cavalcanti A. B. (2021). Ventilatory variables and mechanical power in patients with acute respiratory distress syndrome. *Am. J. Respir. Crit. Care Med.* Available online at: https://www.atsjournals.org/doi/abs/10.1164/rccm.202009-3467OC (accessed April 6, 2021). 10.1164/rccm.202009-3467OC 33784486

[B13] Cressoni M. Gotti M. Chiurazzi C. Massari D. Algieri I. Amini M. (2016). Mechanical Power and development of ventilator-induced lung injury. *Anesthesiology* 124 1100–1108. 26872367 10.1097/ALN.0000000000001056

[B14] Dreyfuss D. Saumon G. (1998). Ventilator-induced lung injury: lessons from experimental studies. *Am. J. Respir. Crit. Care Med.* 157 294–323. 9445314 10.1164/ajrccm.157.1.9604014

[B15] Gattinoni L. Caironi P. Pelosi P. Goodman L. R. (2001). What has computed tomography taught us about the acute respiratory distress syndrome? *Am. J. Respir. Crit. Care Med.* 164 1701–1711. 10.1164/ajrccm.164.9.2103121 11719313

[B16] Gattinoni L. Tonetti T. Cressoni M. Cadringher P. Herrmann P. Moerer O. (2016). Ventilator-related causes of lung injury: the mechanical power. *Intens. Care Med.* 42 1567–1575. 10.1007/s00134-016-4505-2 27620287

[B17] Giosa L. Busana M. Pasticci I. Bonifazi M. Macrì M. M. Romitti F. (2019). Mechanical power at a glance: a simple surrogate for volume-controlled ventilation. *Intens. Care Med. Exp.* 7:61. 10.1186/s40635-019-0276-8 31773328 PMC6879677

[B18] Grommes J. Soehnlein O. (2011). Contribution of neutrophils to acute lung injury. *Mol. Med.* 17 293–307. 21046059 10.2119/molmed.2010.00138 PMC3060975

[B19] Güldner A. Braune A. Ball L. Silva P. L. Samary C. Insorsi A. (2016). Comparative effects of volutrauma and atelectrauma on lung inflammation in experimental acute respiratory distress syndrome. *Crit. Care Med.* 44 e854–e865. 10.1097/CCM.0000000000001721 27035236 PMC5105831

[B20] Guttmann J. (2010). HS-404.2 Energietransfer Beatmungsgerät-Patient – Kann man das weiter minimieren? *Anästh Int.* 51:527.

[B21] Hedenstierna G. Lundquist H. Lundh B. Tokics L. Strandberg A. Brismar B. (1989). Pulmonary densities during anaesthesia. An experimental study on lung morphology and gas exchange. *Eur. Respir. J.* 2 528–535. 2744136

[B22] Huhle R. Serpa Neto A. Schultz M. J. Gama de Abreu M. (2018). Is mechanical power the final word on ventilator-induced lung injury?-no. *Ann. Transl. Med.* 6 394. 10.21037/atm.2018.09.65 PMC6212365 30460268

[B23] Jones H. A. Sriskandan S. Peters A. M. Pride N. B. Krausz T. Boobis A. R. (1997). Dissociation of neutrophil emigration and metabolic activity in lobar pneumonia and bronchiectasis. *Eur. Respir. J.* 10 795–803. 9150315

[B24] Kano S. Lanteri C. J. Duncan A. W. Sly P. D. (1994). Influence of nonlinearities on estimates of respiratory mechanics using multilinear regression analysis. *J. Appl. Physiol.* 77 1185–1197. 10.1152/jappl.1994.77.3.1185 7836121

[B25] Kiss T. Bluth T. Braune A. Huhle R. Denz A. Herzog M. (2019). Effects of positive end-expiratory pressure and spontaneous breathing activity on regional lung inflammation in experimental acute respiratory distress syndrome. *Crit. Care Med.* 47 e358–e365. 30676338 10.1097/CCM.0000000000003649 PMC6433156

[B26] Lanteri C. J. Kano S. Sly P. D. (1994). Validation of esophageal pressure occlusion test after paralysis. *Pediatr. Pulmonol.* 17 56–62. 10.1002/ppul.1950170110 8108177

[B27] Marini J. J. Jaber S. (2016). Dynamic predictors of VILI risk: beyond the driving pressure. *Intens. Care Med.* 42 1597–1600. 10.1007/s00134-016-4534-x 27637717

[B28] Matthay M. A. Zemans R. L. (2011). The acute respiratory distress syndrome: pathogenesis and treatment. *Annu. Rev. Pathol.* 6 147–163. 20936936 10.1146/annurev-pathol-011110-130158 PMC3108259

[B29] Mead J. Takishima T. Leith D. (1970). Stress distribution in lungs: a model of pulmonary elasticity. *J. Appl. Physiol.* 28 596–608. 10.1152/jappl.1970.28.5.596 5442255

[B30] Meade M. O. Cook D. J. Guyatt G. H. Slutsky A. S. Arabi Y. M. Cooper D. J. (2008). Ventilation strategy using low tidal volumes, recruitment maneuvers, and high positive end-expiratory pressure for acute lung injury and acute respiratory distress syndrome: a randomized controlled trial. *JAMA* 299 637–645. 18270352 10.1001/jama.299.6.637

[B31] Mercat A. Richard J.-C. M. Vielle B. Jaber S. Osman D. Diehl J.-L. (2008). Positive end-expiratory pressure setting in adults with acute lung injury and acute respiratory distress syndrome: a randomized controlled trial. *JAMA* 299 646–655. 18270353 10.1001/jama.299.6.646

[B32] Musch G. Venegas J. G. Bellani G. Winkler T. Schroeder T. Petersen B. (2007). Regional gas exchange and cellular metabolic activity in ventilator-induced lung injury. *Anesthesiology* 106 723–735. 10.1097/01.anes.0000264748.86145.ac 17413910

[B33] Pelosi P. Rocco P. R. M. Gama de Abreu M. (2018). Close down the lungs and keep them resting to minimize ventilator-induced lung injury. *Crit. Care* 22:72. 10.1186/s13054-018-1991-3 29558993 PMC5861643

[B34] Rich P. B. Douillet C. D. Hurd H. Boucher R. C. (2003). Effect of ventilatory rate on airway cytokine levels and lung injury. *J. Surg. Res.* 113 139–145. 10.1016/S0022-4804(03)00195-1 12943823

[B35] Saha D. Takahashi K. de Prost N. Winkler T. Pinilla-Vera M. Baron R. M. (2013). Micro-autoradiographic assessment of cell types contributing to 2-deoxy-2-[(18)F]fluoro-D-glucose uptake during ventilator-induced and endotoxemic lung injury. *Mol. Imag. Biol.* 15 19–27. 10.1007/s11307-012-0575-x 22752654 PMC6391052

[B36] Sassoon C. S. H. Mahutte C. K. (1998). *Work of Breathing During Mechanical Ventilation: Physiological Basis of Ventilatory Support.* New York, NY: Marcel Dekker Inc., 261–310.

[B37] Schroeder T. Melo M. F. V. Venegas J. G. (2011). Analysis of 2-[Fluorine-18]-Fluoro-2-deoxy-D-glucose uptake kinetics in PET studies of pulmonary inflammation. *Acad. Radiol.* 18 418–423. 10.1016/j.acra.2010.11.019 21292507 PMC3114040

[B38] Serpa Neto A. Deliberato R. O. Johnson A. E. W. Bos L. D. Amorim P. Pereira S. M. (2018). Mechanical power of ventilation is associated with mortality in critically ill patients: an analysis of patients in two observational cohorts. *Intens. Care Med.* 44 1914–1922. 10.1007/s00134-018-5375-6 30291378

[B39] Torigian D. Chong E. Schuster S. Hofheinz F. Rosenbaum J. Alavi A. (2009). Feasibility and utility of ROVER software for 3D quantitative image analysis of FDG-PET in patients with diffuse large B-cell lymphoma (DLBCL). *J. Nuc. Med.* 50(Suppl. 2) 135–135.

[B40] Vassalli F. Pasticci I. Romitti F. Duscio E. Aßmann D. J. Grünhagen H. (2020). Does Iso-mechanical Power lead to iso-lung damage?: an experimental study in a porcine model. *Anesthesiology* 132 1126–1137. 10.1097/ALN.0000000000003189 32032095

[B41] Wadsak W. Mitterhauser M. (2010). Basics and principles of radiopharmaceuticals for PET/CT. *Eur. J. Radiol.* 73 461–469. 10.1016/j.ejrad.2009.12.022 20181453

[B42] Wittenstein J. Scharffenberg M. Braune A. Huhle R. Bluth T. Herzog M. (2020). Effects of variable versus nonvariable controlled mechanical ventilation on pulmonary inflammation in experimental acute respiratory distress syndrome in pigs. *Br. J. Anaesth* 124 430–439. 10.1016/j.bja.2019.12.040 32033744 PMC8016484

[B43] Zemans R. L. Colgan S. P. Downey G. P. (2009). Transepithelial migration of neutrophils: mechanisms and implications for acute lung injury. *Am. J. Respir. Cell Mol. Biol.* 40 519–535. 10.1165/rcmb.2008-0348TR 18978300 PMC2677434

